# Identification of a novel non-sense mutation in *TBX5 * gene in pediatric patients with congenital heart defects

**DOI:** 10.15171/jcvtr.2018.07

**Published:** 2018-03-17

**Authors:** Mehri Khatami, Mohammad Mehdi Heidari, Fatemeh Kazeminasab, Razieh Zare Bidaki

**Affiliations:** Department of Biology, Faculty of Science, Yazd University, Yazd, Iran

**Keywords:** Congenital Heart Diseases, TBX5, Non-sense Mutation, T-Box Domain, PCR-SSCP

## Abstract

***Introduction:*** Congenital heart diseases (CHDs) are structural cardiovascular malformations that arise from abnormal development of the heart during the prenatal life. Mutations in the *TBX5 * gene, encoding T-box transcription factor, are a major cause of CHD. To evaluate the *TBX5 * mutations in hotspot exons in sporadic pediatric patients with CHD phenotypes, analytical case/control study performed in an Iranian cohort of unrelated patients with clinical diagnosis of congenital heart malformations.

***Methods: ***We investigated TBX5 coding exons 4, 5, 6 and 7 in 95 sporadic patients with CHD phenotypes and compared to 82 healthy controls using PCR-SSCP and DNA sequencing approaches.

***Results:*** We report here on a novel and heterozygote Non-sense mutation in exon 5 of *TBX5*, E128X (G14742T), in two Iranian children. This mutation locates inside the T-box and both of pediatric patients carrying this novel mutation suffer from severe heart malformations. The G14742T mutation leads to the substitution of glutamic acid (E) by stop codon (X) at residue 128, an evolutionarily conserved position in T-box as well as in other species. The non-sense mutation of E128X was predicted to be pathogenic by Mutation Taster and Polyphen software programs.

***Conclusion: ** TBX5* E128X mutation results in a translational premature stop. This type of mutation results in a shortened protein that may function improperly and which cannot bind to other transcription factors; therefore, haploinsufficiency of *TBX5* protein is presumably causing the severe cardiac malformations in these patients.

## Introduction

Congenital heart diseases (CHDs) are the most common defects in heart formation which are significant causes of neonatal morbidity and mortality, with a worldwide incidence of 7 per 1000 live births.^1^ The CHD etiology is complex and is connected with both genetic and environmental reasons. Moreover, CHD has genetically heterogeneous symptoms and despite the molecular progress in clinical diagnosis and our understanding of disease, the causes and mechanisms of CHD remain largely elusive. Heart development is a complex progression that needs accurate interactions of signaling molecules, transcription factors and related co-factor proteins.^2^ During the last decades, candidate-gene screening and linkage analysis have facilitated to recognize the genetic factors in CHD. With the development of whole exome/genome sequencing using the next-generation sequence technology (NGS), it is very possible that a lot of CHD causing genes will be recognized. Cardiac transcription factors are significance determinants of cardiomyocyte differentiation and heart development; therefore, nucleotide changes in the genes encoding these factors are a main cause of CHD.^3,4^ The main of transcription factors, containing the GATA family zinc finger proteins; homeodomain protein NKX2-5; MADS or SRF box proteins and MEF2 factors; T-box transcription factors (including TBX1, TBX2, TBX3, TBX5, TBX18, and TBX20); and Isl1 protein (Lim-homeodomain protein), are very important for control embryonic cardiac development such as cardiac morphogenesis, conduction system progress, heart chamber maturation and endocardial reformation.^5-7^ Therefore, it is not surprising that mutations in the genes encoding the critical heart transcription factors are linked with CHD. Most mutations in regulatory regions of *TBX5* gene can cause protein defects such as impairment in transcriptional activity, DNA binding and or protein-protein interactions.^8,9^ So far, nearly 100 nucleotide changes in this gene have been recognized from CHD patients. Although CHD related mutations are dispersed across all the coding exons of *TBX5* gene, the most of them are positioned within the highly evolutionary conserved DNA binding motif (T-box or T-domain), spanning from amino acids 53–241.^10,11^ Loss-of function mutations in T-box region on the human chromosome 12 (12q24.1) and haploinsufficiency of TBX5 are the cause of CHD. Such nucleotide alterations in T-box affect interactions with the major and minor groove of the target DNA and produce very important heart defects in the developmental pathways of cardiac morphogenesis.^12,13^ In this case/control study, to evaluate the somatic *TBX5* mutations in hotspot exons, we have screened 95 sporadic Iranian pediatric patients with CHD phenotypes and compared to 82 healthy matched controls using PCR-SSCP and DNA sequencing approaches.

## Materials and Methods

### Study population

We performed TBX5 mutation analysis in a cohort of unrelated pediatric patients with clinical diagnosis of congenital heart malformations referred to Afshar cardiac hospital (Yazd, Iran). All patients were clinically evaluated by a pediatric cardiologist. History and physical examination, 12-lead electrocardiogram and echocardiography were performed by experienced Clinicians. In the patient group, 62 (65.27%) individuals had ventricular septal defect (VSD) and 25 (26.31%) suffered from atrial septal defects (ASDs). Also Tetraology of Fallot (TOF) was identified in 8 (8.42%) patients. Sixty-two were female (65.27%) and 33 were male (34.73%). The median age of our patients at time of surgery was 2.3 years (range 0- 3.8 years). In addition, 82 unrelated healthy individuals served as the control (38 male and 44 female). They had normal echocardiographic param­eters without any signs of cardiac disease and family history of CHD. Written informed consent was obtained from all participants and 5 mL of peripheral blood sample was collected from all of cases. Genomic DNA was extracted from peripheral lymphocytes using DNA isolation kit (Cinnagen Co, Tehran, Iran).

### Genotyping

*TBX5* coding exons 4, 5, 6 and 7 together with adjacent boundary regions were amplified using PCR (polymerase chain reaction) with specific primers (Table 1). The in silico primers accuracy for checking their efficiency and specificity, can be performed using BLAST tool and Gene Runner software. PCR reaction was carried out in a 25 µl mixture containing 1×PCR buffer, 50 ng genomic DNA as the template, 10 pmol from each forward and reverse primers, 1.7 mmol/L MgCl_2_, 0.2 mmol/L in each dNTPs and 3 U Taq DNA polymerase (Roche). The amplification program for the cycle reactions were performed in a thermocycler with a three-step PCR protocol: An preliminary denaturation phase at 94°C for 5 minUTES, followed by 35 cycle at 94°C for 35 seconds; 57-63.5°C for 45 seconds (due to different primers); 72°C for 50 seconds and a finishing extension at 72°C for 7 minutes. The PCR products were examined with 1.5% agarose gel electrophoresis and stained with ethidium bromide. For mutation screening, SSCP analysis was performed in all of cases. In SSCP analyze, PCR products were denatured at 95ºC for 10 minutes and then chilled on ice for 5 minutes and loaded on to a non-denaturing 6% polyacrylamide/TBE 0.5x gel containing with 10% glycerol. The gel electrophoresis under the following conditions: 12 hours, 200 V, room temperature and 40 mA. After the running phase, the gel was stained with silver staining, and analyzed. Any changes in electrophoretic migration in single strand DNA bands were analyzed by comparison with normal healthy cases and sequenced by Macrogene agency, South Korea.

**Table 1:** The Primers Used for Amplification of hotspot exons in *TBX5* gene

**Exon**	**Primer sequences**	**Nucleotide positions**
Exon4	F: 5'-AACGGGGCTAGTTTCCGCTTCCACG-3'	8685-8709
	R:5'-CTTTTTGGGAGAAGGTTCCACTTTTC-3'	8966-8991
Exon5	F: 5'-ACCTGGTGCGTGAACTGAAG-3'	14634-14653
	R: 5'-GAGGACAAGAGGGAGACAAG-3'	14898-14917
Exon6	F: 5'- GCAAAAGAAAGAGCAGACGG-3'	18489-18508
	R: 5'- TTATCTGGAGACAAAGGGAG -3'	18736-18755
Exon7	F: 5'- GAGGAGAAAGTTGAGGAATC-3'	27827-27846
	R: GCAGCAATCAAGTGAAAACC-3'	27979-27998

### In silico analysis (bioinformatics techniques)

The Blast analysis and online multiple sequence alignment ClustalW2 (http://www.ebi.ac.uk/tools/msa/clustalw2) was utilized to find the degree of sequence homology that has been acquired in this research and with other sequences of the different species. Also, PolyPhen-2 (http://genetics.bwh.harvard.edu/pph2/) was used for determine the effect of the newly discovered TBX5 mutation on protein sequence. The prediction of poten­tial pathogenicity of a new *TBX5* sequence variation was done by Mutation Taster (http://www.mutationtaster.org), which automatically yielded a possibility for the nucleotide chain to be either a pathogenic mutation or a benign poly­morphism.

## Results

**Figure 1:**
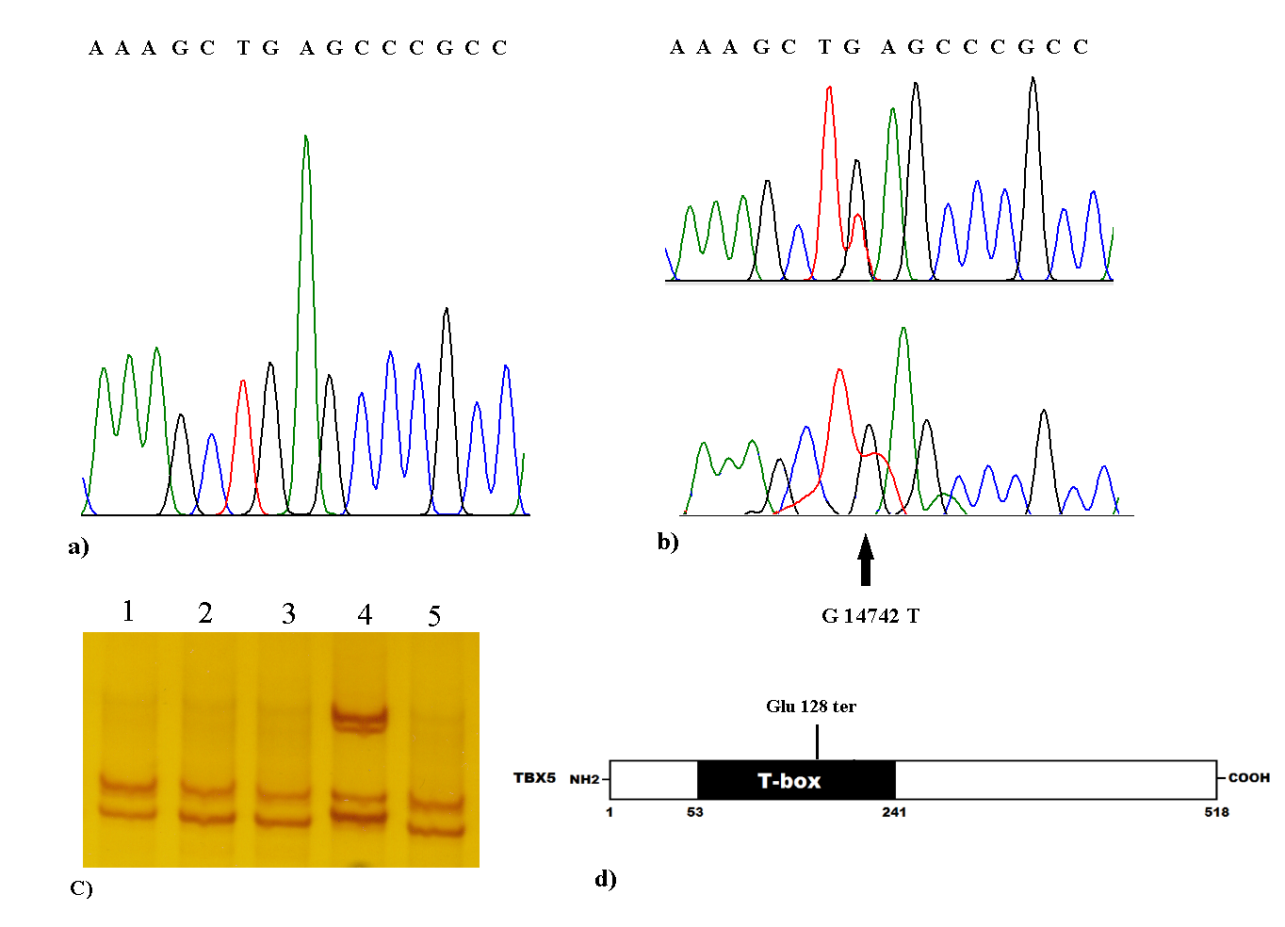
Novel non-sense TBX5 mutation associated with congenital heart defects. (A) Sequence chromatogram showing the normal sequence of exon 5 in TBX5 gene. (B) G to T heterozygote transition (G14742) E128X in the same region in two patients. The arrow indicates the heterozygous nucleotides of G/T in the two mutation carriers. (C) SSCP results. Lanes 1 shows a normal healthy control, lanes 2, 3 and 5 show patients who had not any mutations and lane 4 shows a patient with a heterozygous C14742T mutation (D) Schematic diagram of TBX5 protein structures with the identified mutation marked in T-box domain. NH2 denotes amino-terminus, and COOH, carboxyl-terminus.

**Figure 2:**
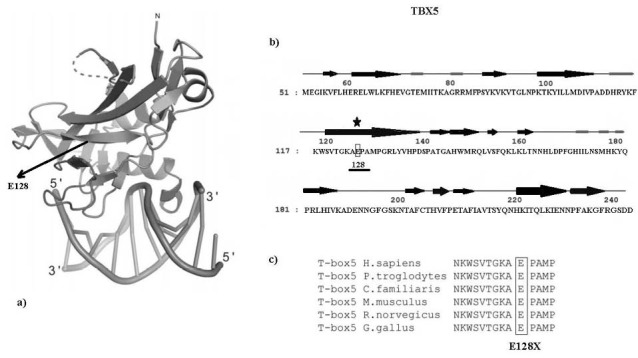
(a) Structural schematic of TBX5-DNA complex. E128X mutation presented with arrow. (b) Amino acid sequence of T-box domain in TBX5 protein. Β-strands depicted as arrows and c) an alignment of amino acid sequences of a segment of TBX5 protein in different species.

To determine whether TBX5 probably disease-causing variants in exons 4, 5, 6 and 7 could occur with CHD, we recruited 95 CHD patients for genetic screening. Among all unrelated patients, for first time we found a novel and heterozygous non-sense putative mutation (a G→T heterozygote transition at nucleotide 14742) in the exon 5 of *TBX5* gene, in two patients (1 and 3 months old infants) with atrial septal defects (ASD) and large ventricular septal defects. These children died during surgery. This heterozygous change, G14742T in exon 5 of TBX5, is leading to a substitution of Glutamic acid at amino acid position 128 for a premature stop codon (p.E128X). This putative mutation has not been reported previously in any literature and not presented in dbSNP and 1000G exome data. The parents of two pediatric patients had a normal clinical features, normal ECG and echocardiography, and did not smoke. They also had non-consanguineous marriage and had not positive family history of CHD, therefore, this novel putative mutation (p.E128X), may be has occurred de novo. The sequencing results presenting the heterozygous *TBX5* nucleotide variation in contrast to its corresponding normal sequence are showed in Figure 1. This non-sense putative mutation was not founded in the 82 healthy subjects and also it was not observe in the SNP databases, suggesting a novel putative mutation in CHD patients. Alignment of multiple TBX5 protein sequences across species is presented that the glutamic acid at amino acid position 128 of human T-box, the DNA-binding domain, was highly conserved evolutionarily among various species (Figure 2), suggesting that this amino acid is functionally very significant.

The non-sense putative mutation of E128X in exon 5 of *TBX5 *was predicted to be pathogenic by Mutation Taster, with a score of 0.99965. No polymorphisms and normal variations in the mutated region were informed in the Mutation Taster database. This sequence variation (G14742T) was also predicted to has a damaging effect by Polyphen software program, PolyPhen-2, with a score of 0.997 (sensitivity, 0.49; specificity, 0.96).

## Discussion

In this research, we present a novel non-sense putative mutation (E128X) which is associated with CHD in two patients with heart defects. Our results show that this TBX5 mutation is predicted to lead to a premature translational stop and truncated gene product in the mutation carriers. The effect of this putative mutation is yet unknown, but it is proposed to cause CHD. Both carriers of this mutation died during surgery that indicates the severe phenotype of atrial septal defects (ASD) and ventricular septal defects (VSD). Both of them were male and CHD was diagnosed by echocardiography (ECG) at age 1 months and 3 months and none of their mothers during pregnancy not exposed to drugs, smoking, alcohol, or infections. One of those two patients has complex appearances, ASD with VSD, and another one has isolated VSD. This putative mutation (G14742T) locates within the T-box domain and both of pediatric patients carrying this novel variation suffer from severe heart defects. In this study, clinical appearances of congenital heart defects in our patients mostly was malformations of the upper limbs, cardiac defects presented contain isolated VSD and ASD and also TOF, associated with impairment of normal cardiac conduction system development. In most cases, the TBX5 mutations result in a loss-of-function manner and only a few of them present a gain-of-function effect.^14-18^ TBX5 E128X mutation results in a translational premature stop, and encodes a non-functional abolished protein, which is probably degraded owing to nonsense mediated decay (NMD) mechanisms and therefore represents individuals with functional haploinsufficiency. This type of mutations may be more informative due to the importance of particular residues or active domains within the protein, and or determine of novel functions of the protein domains. The mainly functional activity of TBX5 detects within the DNA-binding T-BOX domain which spans residues 53 to 241. The E128X putative mutation approximately is positioned in central area (Figure 2a). Previous interaction researches using electrophoretic mobility shift analysis presented that the DNA-binding T-BOX domain of TBX5 is critical for interaction with NKX2.5 and GATA4 whereas the other regions are not.^19^ Schott et al. and Ghosh et al. argued that the most of the mutations in *TBX5* gene are affective in DNA binding, protein-protein interactions and transcriptional activity.^20,21^ Other related researches also showed the important role of TBX5 in various types of CHDs.^22^ To date more than one hundred mutations in this gene have been recognized from patients with CHD.^23^

According to RCSB protein data bank, The E128 residue is located in coil region between the two areas of bete strands of the T-box (Figure 2b), which is in adjacent proximity to the DNA and may be leads to a comprehensive loss of DNA binding. So probably, the mutant protein cannot bind to other transcription factors such as NKX2-5 and GATA4 and accordingly, this defective protein acts like a nonfunctional variant. Therefore, haploinsufficiency of correct TBX5 protein is presumably causing the severe cardiac malformations seen in these patients. Moreover, mutation prediction programs (Polyphen and Mutation Taster) ruled out a possible damaging effect of this protein variant. It is predicted that E128X mutation creates a premature stop codon resulting in the production of a truncated protein or no protein in cardiac cells as a result of nonsense-mediated mRNA decay. However, this putative mutation has not previously been described and its functional effects remain unclear. Interestingly, the nucleotide sequences in these regions of *TBX5* gene is highly GC rich, which may have provided to the various independent nucleotide changes in these exons as mutation hotspots.^24^ Since amino acids 1-237 of TBX5 are essential for DNA binding, any mutations in this area is predicted to disrupt binding of TBX5 transcription factor to the major and minor groove of DNA and substitution of glutamic acid residue to a stop codon in E128X is surely expected to be disruptive. In early studies, Basson et al. identified a nonsense mutation (Glu69X) in affected members of one family that produced a truncated protein without a T-box domain, and in Chinese patients, Yang et al reported three novel variations that included a deletion of one base pair with frame shift effect, and two non-synonymous mutations.^25,26^ The results showed that missense and non-sense mutations in T-BOX mainly induce more complex cardiac malformations and sever heart defects in CHD patients.

To our knowledge, in earlier protein interaction analysis using in vitro translated TBX5 mutants, the G125R, R237W, R237P and I106V mutations lead to impaired nuclear localization and binding to cardiac-specific interaction partners such as NKX2-5 and GATA4.^27^ Their results showed loss of DNA-binding and abolish of interaction with specific protein partners leading to decreased transcription activation of certain TBX5 target genes so these mutations in T-BOX result in a functional haploinsufficiency.

## Conclusion

This research represents a novel non-sense putative mutation which is associated with CHD. Our findings in this study are very important because these new observations increase our knowledge of the spectrum of nucleotide changes that lead to CHD and also generate interesting questions about the genetic heterogeneity of these heart defects and their mutations.

## Study limitations

The major limitations of this study were the relatively small sample size of enrolled patients and lack of long term follow-up and missing of entire medical records after case sampling. Also, other segments of *TBX5* gene (i.e. promoter region) are not evaluated in this research.

## Ethical Approval

This study was approved by the Yazd University Ethics Committee.

## Competing interests

Authors declare no conflict of interest in this research.
